# Targeted design of synthetic enhancers for selected tissues in the *Drosophila* embryo

**DOI:** 10.1038/s41586-023-06905-9

**Published:** 2023-12-12

**Authors:** Bernardo P. de Almeida, Christoph Schaub, Michaela Pagani, Stefano Secchia, Eileen E. M. Furlong, Alexander Stark

**Affiliations:** 1 Research Institute of Molecular Pathology (IMP), Vienna BioCenter (VBC), Vienna, Austria; 2 Vienna BioCenter PhD Program, Doctoral School of the University of Vienna and Medical University of Vienna, Vienna, Austria; 3 European Molecular Biology Laboratory (EMBL), Genome Biology Unit, Heidelberg, Germany; 4 Medical University of Vienna, Vienna BioCenter (VBC), Vienna, Austria; 5 Present Address: InstaDeep, Paris, France

**Keywords:** Gene regulation, Machine learning

## Abstract

Enhancers control gene expression and have crucial roles in development and homeostasis^1–3^. However, the targeted de novo design of enhancers with tissue-specific activities has remained challenging. Here we combine deep learning and transfer learning to design tissue-specific enhancers for five tissues in the *Drosophila melanogaster* embryo: the central nervous system, epidermis, gut, muscle and brain. We first train convolutional neural networks using genome-wide single-cell assay for transposase-accessible chromatin with sequencing (ATAC-seq) datasets and then fine-tune the convolutional neural networks with smaller-scale data from in vivo enhancer activity assays, yielding models with 13% to 76% positive predictive value according to cross-validation. We designed and experimentally assessed 40 synthetic enhancers (8 per tissue) in vivo, of which 31 (78%) were active and 27 (68%) functioned in the target tissue (100% for central nervous system and muscle). The strategy of combining genome-wide and small-scale functional datasets by transfer learning is generally applicable and should enable the design of tissue-, cell type- and cell state-specific enhancers in any system.

## Abstract (web summary)

Deep learning and transfer learning were used to design tissue-specific enhancers in the *Drosophila* embryo that were active and specific, validating this approach to achieve tissue-, cell type- and cell state-specific expression control.

## Main

Enhancers are non-coding DNA elements that activate transcription from target promoters in a highly cell type-specific fashion^1^. Although the existence of enhancer activities within DNA sequences has been recognized since the early 1980s^2^, and hundreds of enhancers have been functionally characterized in model organisms such as flies^4^ and mice^5^, the precise encoding of regulatory activities within the DNA sequence has remained elusive. Specifically, although it is known that enhancer sequences contain binding sites for transcription factors, the specific arrangement of these sites and the potential role of additional sequence properties have remained unknown, hampering the prediction and the de novo design of enhancers with tissue-specific activities.

By utilizing genome-wide enhancer activity datasets in a model cell line, it is possible to train deep learning convolutional neural networks (CNNs) to predict enhancer activity and strength directly from the DNA sequence and to design synthetic enhancers de novo^6^. However, extending this achievement to in vivo systems has been challenging, presumably owing to the limited number of functionally characterized enhancers, which has remained relatively low, typically falling below a few hundred per tissue in flies^4^ and mice^5^. Such quantities have been considered insufficient for effectively training deep learning models.

A widely applicable approach to enhance prediction performance with limited data is through the utilization of transfer learning, which has been used successfully in various fields^7^, including cell biology^8^, network biology^9^ and genomics^10–13^. Transfer learning involves pre-training models using large-scale datasets that share similarities with the target task, followed by target task-specific adjustment or fine-tuning on smaller datasets. Provided pre-training is carried out with datasets sufficiently similar to the target task, transfer learning yields improved prediction performance^7^. To predict enhancer activity from the DNA sequence, leveraging genome-wide datasets of enhancer-associated chromatin features as a steppingstone seems particularly promising (see, for example, refs. ^3,11,13,14^).

**Fig. 1:**
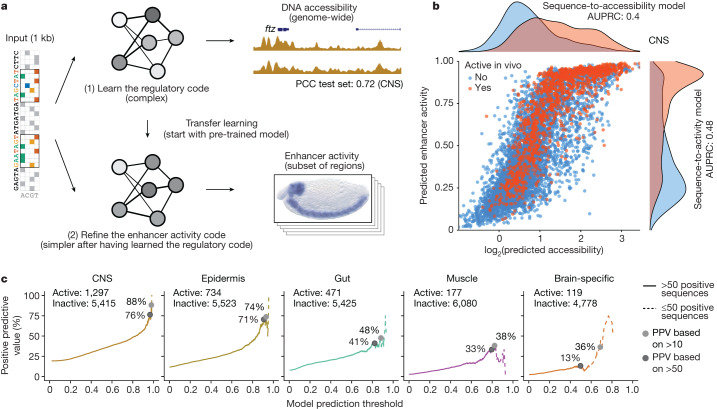
Deep learning-based design of tissue-specific synthetic enhancers. **a**, Overview of the deep and transfer learning strategy for predicting in vivo enhancer activity. First, a CNN is trained to predict quantitative DNA accessibility (pseudo-bulk scATAC-seq data) from the DNA sequence (sequence-to-accessibility model). Shown is a locus from the held-out test chromosome with observed and predicted values for CNS, with a PCC of 0.72. The first model is used to initialize a second model to classify DNA sequences on the basis of their activities in vivo in the respective tissue (sequence-to-activity model; shown is an enhancer active in CNS). This process is done separately for each tissue. **b**, Comparison of predicted DNA accessibility from the sequence-to-accessibility model and predicted enhancer activity (probability) from the sequence-to-activity model in the CNS for all sequences tested in vivo using tenfold cross-validation (blue, inactive; red, active). Density plots show the respective distributions. Area under the precision-recall curve (AUPRC) values are shown for both models. **c**, PPV of enhancer activity predictions at different thresholds. For each threshold (*x* axis, 0–1), the percentage of active sequences among all positive predictions is shown (*y* axis). Solid lines indicate percentages calculated based on more than 50 positive sequences, and dashed lines represent less confident estimates based on smaller numbers.

Single-cell assay for transposase-accessible chromatin with sequencing (scATAC-seq) datasets provide measurements of DNA accessibility at the single-cell level and thus allow the determination of cell type-specific accessibility profiles even within complex tissues comprising diverse cell populations^15^. Given the association of enhancers with accessible chromatin, we decided to use a combination of scATAC-seq datasets and results from in vivo enhancer activity assays to develop a deep learning model predictive of enhancer activity using transfer learning (Fig. 1a).

Specifically, we selected four prominent and distinct tissues within the 10- to 12-hour-old *Drosophila melanogaster* embryo, namely the central nervous system (CNS), epidermis, muscle and gut. In addition, we selected enhancers that were specifically active in the brain but not in the rest of the CNS, an enhancer–activity pattern that we considered particularly challenging given the shared cell types with the CNS and the relatively small number of functionally characterized brain-specific enhancers available for training.

**Extended Data Fig. 1:**
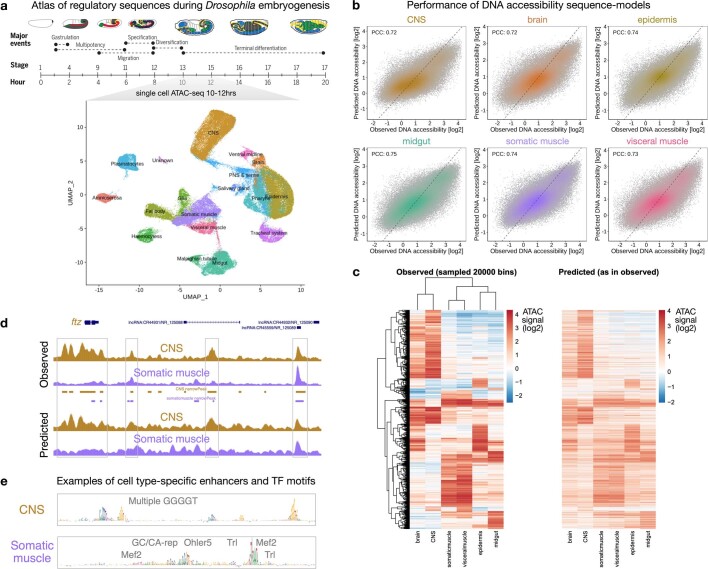
Learning the cis-regulatory code of *Drosophila* embryo tissues with deep learning. **a**) Top: Cartoon with *Drosophila* embryogenesis and respective stages and times, adapted from ref. ^16^. Reprinted with permission from AAAS. Bottom: UMAP visualization of cell-x-peak accessibility matrix of cells with inferred age between 10 and 12 h, colored and labeled by tissue annotation. Data from ref. ^16^. **b**) Performance of sequence-to-accessibility models for the selected pseudo-bulk tissues from (A). Scatter plots of predicted versus observed DNA accessibility signal (units of log depth-normalized coverage) across DNA sequences in the test set chromosomes (downsampled to 100,000 for easier visualization) for each tissue. Color reflects point density. PCC, Pearson correlation coefficient using all DNA sequences. **c**) Heatmaps of observed ATAC signal vs predicted ATAC signal across 20,000 sampled differentially accessible regions. The heatmap with observed values is clustered across regions (rows) and tissues (columns). The heatmap with predicted values has the same row and column orders but colored by the predicted values. **d**) Genome browser screenshot depicting observed and predicted ATAC profiles for the CNS (brown) and somatic muscle (purple) for a locus on the held-out test chromosome. Accessibility peaks for each tissue are shown below the observed signals. High-accessibility regions are highlighted with grey boxes (for example the well-known CNS enhancers upstream of the *ftz* gene). **e**) Nucleotide contribution scores for (top) a CNS and (bottom) a somatic muscle enhancer derived from the respective accessibility models. Instances of TF motifs known to be associated with the respective tissues and predicted to be important for the enhancer activity are highlighted.

**Extended Data Fig. 2:**
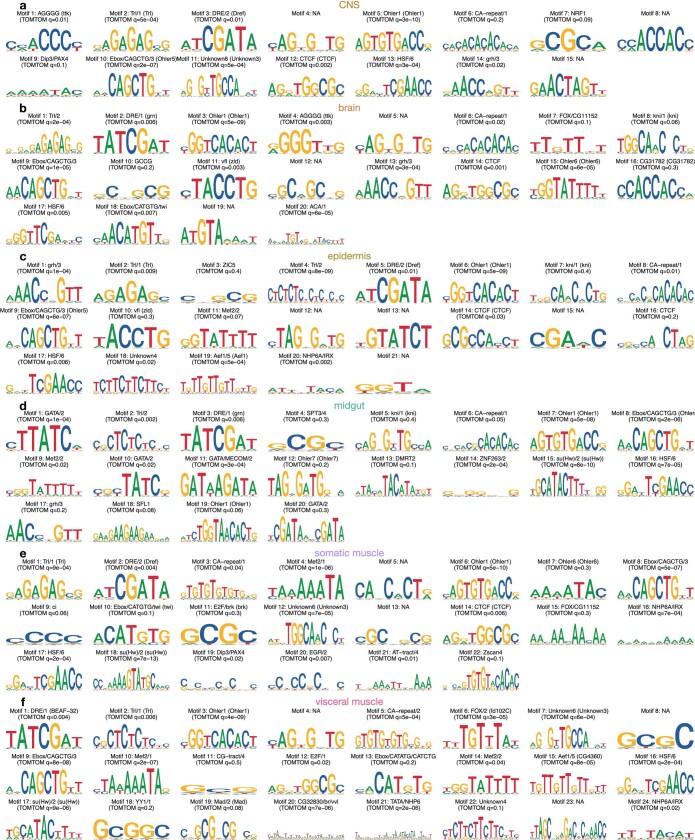
TF motifs predictive of DNA accessibility discovered by TF-Modisco. **a**-**f**) Motifs discovered by TF-Modisco^21–24^ by summarizing recurring predictive sequence patterns from the respective accessible regions of each pseudo-bulk tissue. Motifs are ranked by TF-Modisco predictive value and label by ID (motif number). Shown are the converted PWM logos of each motif, labeled with their closest database match (top: motif cluster (TF name, if available); bottom: PWM ID and TOMTOM q-value^59^). NA means no significant match, based on TOMTOM q-value. See Methods for more details.

We first trained single-task CNNs to map 1-kb-long DNA sequences tiled across the genome to the corresponding pseudo-bulk ATAC-seq signals based on our recently published scATAC-seq atlas of the *Drosophila* embryo^16^ (sequence-to-accessibility models; Fig. 1a and Extended Data Fig. 1a). We used a tenfold chromosome hold-out cross-validation scheme to train and evaluate the predictive performance of the model. As expected on the basis of previous work^6,17–20^, these models performed well with Pearson correlation coefficients (PCCs) between the predicted and experimentally measured ATAC-seq signals of approximately 0.73 for all tissues in all held-out test set chromosomes (range of PCCs: 0.72–0.75; Fig. 1a and Extended Data Fig. 1b,d). Moreover, using model-interpretation tools^21–24^ revealed known transcription factor motifs, such as GGGGT (Kr and Ttk) for CNS^25^, and motifs for Grh for epidermis^26^, GATA for gut^27–29^, Mef2, forkhead (Bin) and Twist for muscle^30^, and Zelda and Klu for brain^31,32^ (Extended Data Figs. 1e and 2, Supplementary Fig. 1 and Supplementary Table 1). Finally, the models also captured cell type-specific differences in accessibility, that is, sites that were preferentially accessible in specific tissues were also predicted to be accessible in these tissues (Extended Data Fig. 1c).

**Extended Data Fig. 3:**
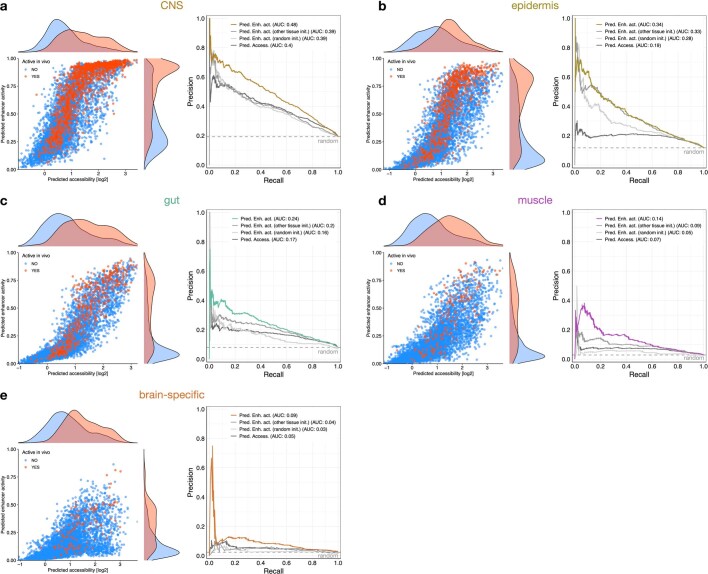
Comparison of sequence-to-accessibility and sequence-to-activity models plus controls. **a**-**e**) Left: Comparison of predicted DNA accessibility [log2] and predicted enhancer activity [probability] in each tissue for all tested sequences in vivo (inactive in blue, active in red). Density plots show the respective distributions for both predictions for inactive and inactive sequences. Right: precision-recall curves for the sequence-to-accessibility and sequence-to-activity models on test data, plus two additional controls: models trained directly on the in vivo enhancer activity data starting from random initialization and models pre-trained on ATAC-seq data from an unrelated tissue (salivary gland). Respective areas under the precision-recall curve (AUC) are shown. Predictions for all models were computed for each sequence only using the respective cross-validation set where the sequence is held-out for testing.

**Extended Data Fig. 4:**
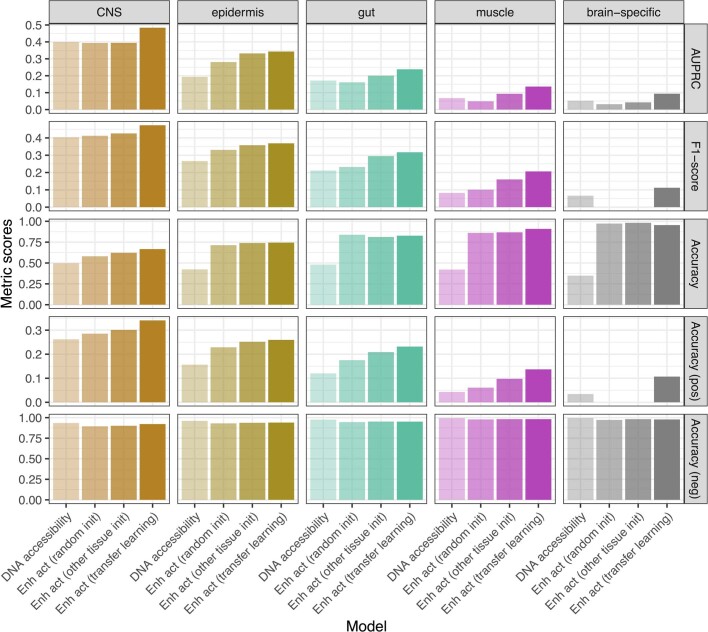
Metric evaluation of the different models. The performance of different models (x-axis) per tissue (column) was evaluated on test data with five different metrics: area under the precision-recall curve (AUPRC), F1-score, accuracy across all sequences, only among positive, or only among negative sequences. The models are the ones from Extended Data Fig. 3: the sequence-to-accessibility (DNA accessibility) and sequence-to-activity (transfer learning) models, plus control models trained directly on the in vivo enhancer activity data starting from random initialization or pre-trained on ATAC-seq data from an unrelated tissue.

**Extended Data Fig. 5:**
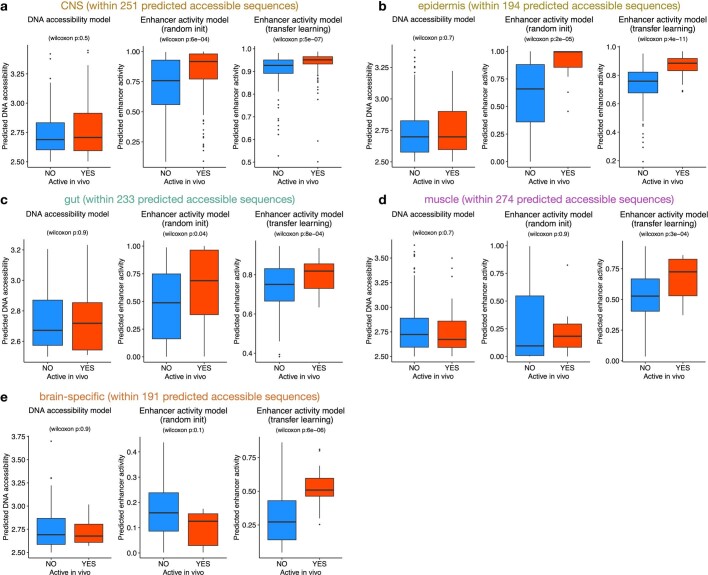
Predictive value of DNA accessibility and enhancer-activity models for predicted accessible sequences. **a**-**e**) For each tissue, sequences in the test set were selected based on a predicted DNA accessibility value higher than 2.5 and scored with the different models (total number of selected sequences shown in panel title). Sequences inactive (blue) or active (red) in vivo are shown in boxplots in function of their scores by the DNA accessibility model, enhancer activity model starting from random initialization, and enhancer activity model using transfer learning. P-values from two-sided Wilcoxon rank-sum test are shown for each comparison between inactive and active sequences. Numbers of predicted accessible sequences used for statistics per tissue: CNS – 251, epidermis – 194, gut – 233, muscle – 274, brain-specific – 191. The boxplots mark the median, upper and lower quartiles and 1.5× interquartile range (whiskers); outliers are shown individually.

**Extended Data Fig. 6:**
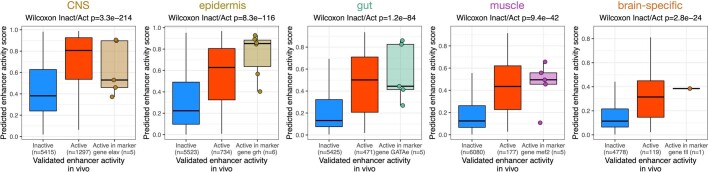
Model evaluation on positive and negative control sequences. Predicted enhancer activity scores by the sequence-to-activity transfer learning models for validated inactive sequences, all known active enhancers, and for known enhancers in the marker gene loci of the respective tissues. Gene loci (+/−50kb): *elav* (CNS), *grh* (epidermis), *GATAe* (gut), *Mef2* (muscle) and *tll* (brain). P-values from two-sided Wilcoxon rank-sum test are shown for each comparison between inactive and active sequences per tissue. Number of sequences in each boxplot is shown in the respective x-axis. The boxplots mark the median, upper and lower quartiles and 1.5× interquartile range (whiskers); outliers are shown individually.

We next utilized functionally characterized enhancers from our previous work^4,33^ for transfer learning to build sequence-to-activity models. We framed the enhancer–activity prediction task as a binary classification (active/inactive) as the in vivo enhancer–activity data are derived from annotated non-quantitative in situ hybridization assays^4,33^. We initialized CNNs to predict tissue-specific enhancer activities directly from the DNA sequence by the sequence-to-accessibility models trained on ATAC-seq data for the respective tissues (CNS, epidermis, gut, muscle and brain—see previous paragraph), and trained an enhancer prediction task until convergence (Fig. 1a; see Methods). We evaluated the models using cross-validation with left-out datasets containing active and inactive enhancers, with and without ATAC-seq signals. This revealed that the sequence-to-activity models obtained by transfer learning substantially improved the predictions for all five tissues as assessed by several performance measures compared to: (1) models directly trained on the in vivo enhancer activity data starting from random initialization; (2) models pre-trained on ATAC-seq data from a different tissue (salivary gland); and (3) the sequence-to-accessibility models without transfer learning (Fig. 1b and Extended Data Figs. 3 and 4). The transfer-learned models also outperformed the other models in correctly discriminating accessible regions with and without enhancer activity, and the improvement was particularly strong for muscle and brain, which had the fewest known enhancers for training (177 and 119, respectively) (Extended Data Fig. 5). The models also reliably discriminated additional positive and negative control enhancers, including the known enhancers in tissue-specific marker gene loci (Extended Data Fig. 6).

**Fig. 2:**
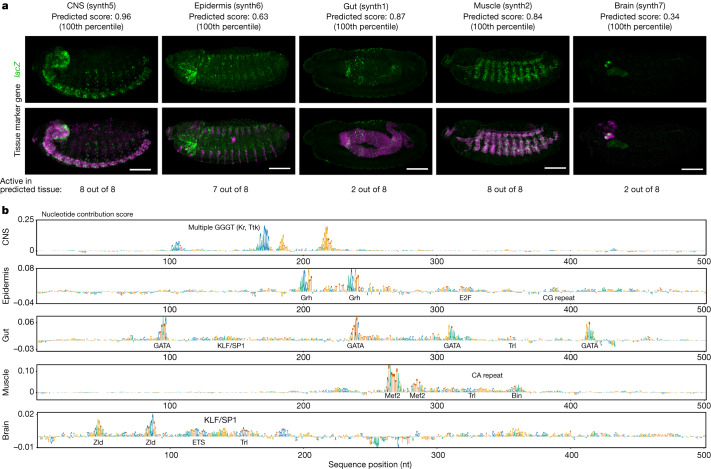
Validation of synthetic enhancers in vivo. **a**, In vivo enhancer activity of one active sequence per tissue, as an example (for all other active sequences, see Extended Data Fig. 9). For each sequence, one representative embryo is shown from the total 200–300 embryos stained with double RNA fluorescence in situ hybridization (FISH). Scale bar, 100 μm. Predicted enhancer activity score and percentile value for the respective tissue model are shown. Top row, *lacZ* intensity reflects enhancer activity. Bottom row, *lacZ* intensity (green) overlaid with an endogenous marker gene (pink) for the respective tissue: *elav* (CNS), *wg* (epidermis), *GATAe* (gut), *Mef2* (muscle) and *tll* (brain). The total numbers of active sequences per tissue are shown. **b**, Nucleotide contribution scores for the synthetic enhancers in **a** derived from the enhancer activity models for the respective tissues using DeepExplainer^22–24^. Instances of transcription factor motifs known to be associated with the respective tissues and predicted to be important for the enhancer activity are highlighted.

**Extended Data Fig. 7:**
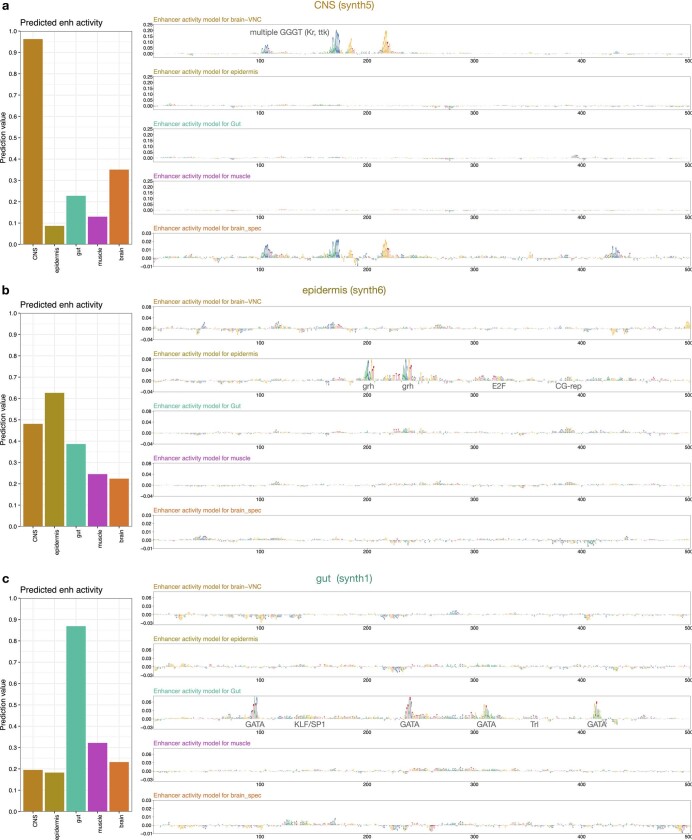
Nucleotide contribution scores of synthetic enhancers. **a**-**c**) Left: Predicted enhancer activity across the five tissues for the synthetic enhancers from Fig. 2a. Right: Nucleotide contribution scores for the synthetic enhancers from Fig. 2a derived from the enhancer activity models of the five tissues, using DeepExplainer^22–24^, with important TF motifs annotated.

**Extended Data Fig. 8:**
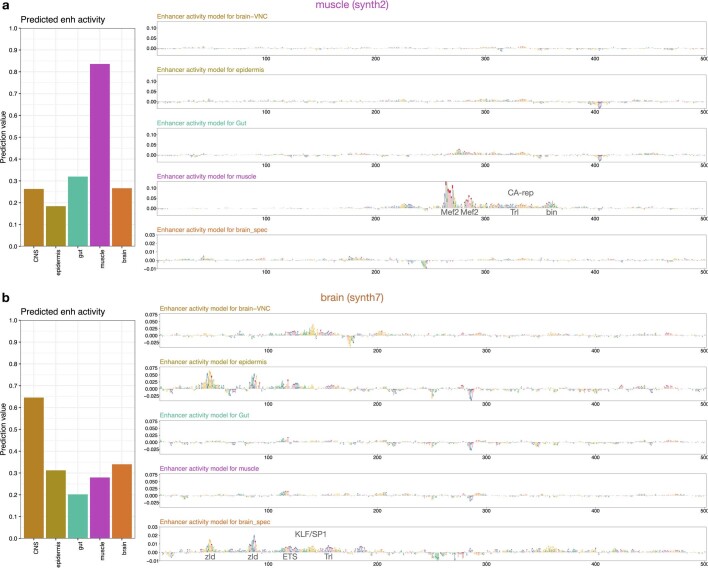
Nucleotide contribution scores of synthetic enhancers. **a**-**b**) Left: Predicted enhancer activity across the five tissues for the synthetic enhancers from Fig. 2a. Right: Nucleotide contribution scores for the synthetic enhancers from Fig. 2a derived from the enhancer activity models of the five tissues, using DeepExplainer^22–24^, with important TF motifs annotated.

Moreover, and particularly relevant for enhancer design that can only test a very limited number of predictions in vivo, these models reached positive predictive values (PPVs) between 36% (brain) to 88% (CNS) at prediction thresholds that recovered at least 10 known enhancers during cross-validation (or PPVs between 13% to 76% at ≥50 known enhancers; Fig. 1c), suggesting that it would not be unreasonable to attempt the de novo design of synthetic enhancers for these tissues. We therefore proceeded to design synthetic enhancers with defined tissue-specific activities de novo (Fig. 2a). Specifically, we created random sequences with a zero-order Markov model and selected 8 enhancers for each of the 5 tissues (40 enhancers total) that had high predicted accessibility and activity scores specifically in the CNS, epidermis, gut, muscle or brain, focusing on distinct motif signatures when possible to remove potential redundancies (see Methods; Extended Data Figs. 7 and 8, Supplementary Fig. 2 and Supplementary Table 2).

We ordered the designed enhancer sequences, cloned them into a previously used reporter system that features a minimal hsp70 promoter and *lacZ* reporter gene, and integrated the constructs into a consistent landing site in the *Drosophila* genome^33^ (see Methods for details on the reporter system and its properties). We then collected and fixed embryos and scored the enhancer activities of the candidates by two-colour fluorescent in situ hybridization, comparing *lacZ* reporter expression to the expression of the tissue-specific marker genes *elav* (CNS), *wg* (epidermis), *GATAe* (gut), *Mef2* (muscle) and *tll* (brain). In addition to a qualitative visual assessment, we also quantitatively compared the expression patterns by pixel-wise PCCs across the entire volumes of the acquired microscopy image *z*-stacks.

**Extended Data Fig. 9:**
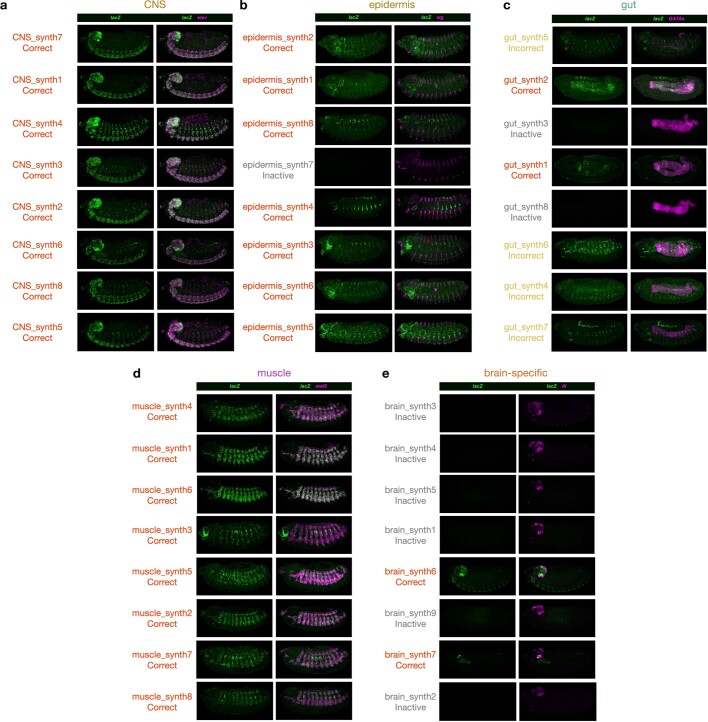
All synthetic sequences experimentally tested as enhancers. **A**-**E**) Left panels show the *lacZ* intensity (green) as a marker for the enhancer activity pattern of the respective candidate sequence (labeled on the left). Right panels show the intensity of both the *lacZ* reporter gene driven by the synthetic sequence (green) and the corresponding endogenous marker gene (pink) for the respective tissue (*elav* (CNS), *wg* (epidermis), *GATAe* (gut), *Mef2* (muscle) and *tll* (brain)). Synthetic enhancers are labeled as correct tissue expression, incorrect tissue expression and inactive. For each sequence, one representative embryo is shown from the total 200–300 double FISH-stained embryos. Scale bar, 100 μm. See Table S2 for more details.

**Extended Data Fig. 10:**
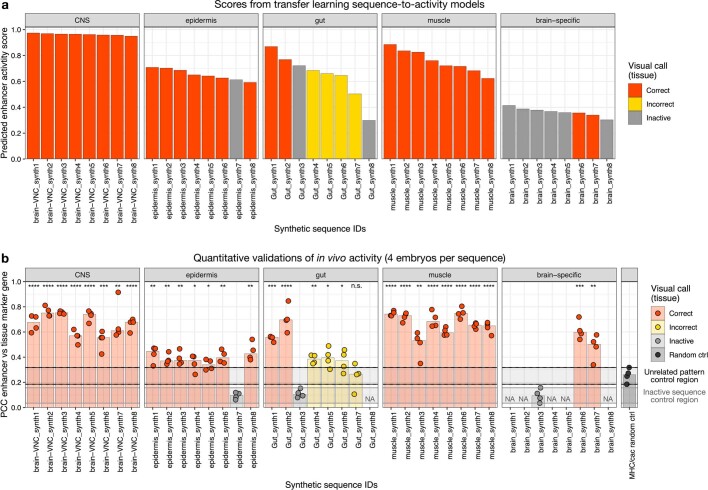
Predicted scores for synthetic sequences and quantitative validations. **a**) Predicted enhancer activity scores by the sequence-to-activity transfer learning models for candidate synthetic enhancers per tissue. Sequences are colored based on their validated in vivo activity: correct tissue expression, incorrect tissue expression and inactive. **b**) Quantitative validations for each candidate synthetic sequence per tissue. Pixel-wise Pearson Correlation Coefficient (PCC) between the marker genes and the synthetic enhancers calculated across the entire embryo volume are shown for 4 embryos per sequence (dots). Barplots represent the respective median value across the 4 embryos. For epidermis, gut, and brain, the PCCs between the marker genes and one inactive candidate per tissue (grey) are displayed. NA: PCCs not quantified for these inactive candidates. As an additional control, PCCs between two unrelated genes are shown (black; see Methods). Sequences are colored based on their validated in vivo activity: correct tissue expression, incorrect tissue expression and inactive. Same order of sequences as in (A). P-values from two-sided t-test between the PCCs of each sequence and the PCCs of two unrelated genes are shown for each sequence: **** p-value < 0.0001, *** <0.001, ** <0.01, * <0.05, n.s. non-significant. The two rectangles represent the interval of PCC values (between minimum and maximum) for the inactive (grey) and unrelated pattern (black) control sequences.

This revealed that eight out of eight CNS enhancers were active in the CNS; some of these had additional, mainly weak and sporadic, activity in the peripheral nervous system (Fig. 2, Extended Data Fig. 9a and Supplementary Table 2). Similarly, seven out of eight epidermis enhancers and eight out of eight muscle enhancers functioned specifically in the epidermis and muscle, respectively (Fig. 2, Extended Data Fig. 9b,d and Supplementary Table 2). For both the gut and brain enhancers, two out of eight were active in the respective target tissue and had partial additional activities in other tissues such as the CNS, salivary gland or amnioserosa (Fig. 2, Extended Data Fig. 9c,e and Supplementary Table 2), in line with the expectations from cross-validation. These results from our qualitative visual assessment were confirmed by quantitative assessment of pattern similarities (Extended Data Fig. 10 and Supplementary Table 2). All patterns deemed correct by visual assessment and three out of the four gut enhancer patterns that were deemed incorrect by visual assessment were significantly different from random and negative control patterns (*t*-test *P* value < 0.05; *n* = 4 embryos).

Notably, given the aim of this study to target broad tissue types that comprise distinct subtypes, not all of the enhancers that were active in the correct target tissue exhibited identical activity patterns. For example, the epidermis enhancers were active in segmental and/or pharyngeal parts of the epidermis, and a similar sub-pattern variability within the correct overall tissue type was seen for CNS and muscle (Extended Data Fig. 9). Also notable are the different success rates for muscle (100%) and gut (25%), and the observation that several gut enhancers were active outside the gut in epidermis, sensory complexes and amnioserosa (Extended Data Fig. 9c and Supplementary Table 2). This probably stems from a more complex gut ‘enhancer grammar’ involving low-information GATA motifs (for example, in Fig. 2c and Extended Data Fig. 2d): the five GATA transcription factors in the fly are utilized rather broadly in endoderm and gut (Serpent and dGATAe^34,35^), but also in amnioserosa, dorsal epidermis, the heart (Pannier^36,37^) and other tissues^38^—that is, the very tissues for which we observe ectopic gut enhancer activity. In this context, it is notable that the pattern similarity (PCC) with the gut marker gene dGATAe is significantly above random for all but one of the gut enhancers deemed incorrect by visual assessment (and for all the correct ones), potentially indicating pattern overlap and/or relatedness of the tissues (Extended Data Figs. 9c and 10b). After this proof of concept at the level of broad tissue types, it will be interesting to see the development of more fine-grained models that discriminate between closely related tissue subtypes and individual cell types, especially those that share prominent transcription factors (such as GATA factors in gut and other tissues).

Overall, our work demonstrates the feasibility of targeted design of synthetic enhancers for selected tissues by deep and transfer learning. The framework proposed here should be applicable to any species and tissue provided a genome-wide dataset of enhancer-associated features (for example, DNA accessibility, characteristic histone modifications, transcription factor or cofactor binding and enhancer RNAs) and a reasonable number of functionally validated enhancers (in this study, more than 100 were used per tissue).

More traditional machine learning approaches have been used successfully for the prediction of chromatin features, transcription factor binding and enhancer sequences^4,39–42^ and for predicting genomic elements with highly constrained *cis*-regulatory codes and limited architectures (for example, core promoter elements^43^ or highly defined enhancer motif contexts^44^). However, the challenge of flexible enhancer design has only become possible with deep learning^6,45^ (and ref. ^46^, which was published as a preprint while this manuscript was under review).

For the near future, we foresee great progress in deep and transfer learning approaches to the prediction and design of enhancers and other genomic regulatory elements. These will probably include the application of large multitask models trained simultaneously on many datasets comprising different tissues and cell types^47^. As predictive sequence features such as transcription factor motifs are often shared between tissues (for example, in Extended Data Fig. 2 and Supplementary Fig. 1), shared learning of large models might further improve model performance compared to the dedicated single-task models used here. Conversely, improved performance might come from the combination of many small, dedicated models such as the ones developed here, each specialized for one specific type of function or genomic element, into a larger overarching framework. Another likely improvement for the specific task of enhancer design will be the move from computational screening of random sequences, which can only sample a very small part of the possible sequence space, to a more direct and efficient way to generate synthetic enhancer sequences, such as the use of generative adversarial networks^48^, variational autoencoders^49,50^ and diffusion models^51^ that can ‘hallucinate’ possible solutions.

Our work complements approaches to design enhancers in or via cell culture models^6,46^ or via the modelling of cell type-characteristic DNA accessibility patterns and their sequence signatures (topic modelling^45^) and ongoing efforts to predict gene expression^47^ and 3D genome architecture^52,53^ from extended DNA sequences. Models to predict endogenous gene expression must integrate the regulatory cues of multiple enhancers acting from different distances, consider distinct promoter types with enhancer–promoter compatibilities, and insulator, silencer and tethering elements, together with the sequence determinants of RNA processing and stability. It will be interesting to see these models integrate lessons from enhancer-centric approaches to further develop and move towards designing entire synthetic gene loci with complex gene-expression patterns.

We envision that our work will synergize with ongoing efforts to build comprehensive ‘cell atlases’ for gene expression and DNA accessibility in the fly, mouse and human, thus providing the opportunity to design enhancers for many, if not all, tissues in these organisms, potentially even for aberrant tissue or cell states. In conclusion, our work not only demonstrates the remarkable progress in enhancer design made possible by deep and transfer learning and the growing datasets on enhancers and chromatin, but also sets the stage for a future in which the precise design and manipulation of gene-expression patterns become a reality.

## Methods

### Processing of pseudo-bulk DNA accessibility data

We retrieved sci-ATAC-seq3 mapped reads (dm6) from each of the 18 tissue pseudo-bulk (that is, mapped reads from all cells combined) at the 10–12 h timepoint from ref. ^16^ (downloaded from https://shendure-web.gs.washington.edu/content/members/DEAP_website/public/ on 1 February 2022, BAM files available upon request; see also Extended Data Fig. 1a). We generated coverage tracks for each tissue pseudo bulks, including the five tissues of interest: CNS, brain, epidermis, midgut and muscle (initially modelled separately for the somatic muscle and visceral muscle pseudo bulks as these were annotated separately in the respective publication, we proceeded with only visceral as explained below). All read fragments from each pseudo-bulk were used for peak calling with MACS2^54,55^ with the following command: macs2 callpeak --nomodel --keep-dup all --extsize 200 --shift −100 --gsize dm -B.

### Deep learning sequence-to-accessibility models

#### Data preparation

We binned the dm6 genome (downloaded from https://hgdownload.soe.ucsc.edu/goldenPath/dm6/bigZips/dm6.fa.gz) into 1,001-bp windows with a stride of 50 bp, and filtered windows in the chromosomes chr2L, chr2R, chr3L, chr3R, chr4, chrX, chrY and chrM. For each window, we computed the log average of the depth-normalized ATAC coverage over the central 201 bp of the window. We combined the accessibility peaks of all scATAC-seq pseudo bulks and selected all bins whose central 151 bp were within any 301 bp-centred peak region. We further added 144,424 random windows throughout the genome with a range of accessibility levels to obtain a dataset with reasonable class imbalances while maintaining high diversity in negative examples. Finally, we only included windows with non-zero ATAC signals across every pseudo-bulk and removed the ones with outlier values (quantile <0.01 or >0.999 in any pseudo-bulk). We augmented our dataset by adding the reverse complement of each original sequence, with the same output, ending up with 464,203 examples (928,406 post-augmentation).

#### Cross-validation scheme

We used a cross-validation scheme to have a more robust model performance. We divided the sequences into ten folds based on their chromosomal positions (considering chromosome halves; see Supplementary Table 3 for the specific folds used) and used a cross-validation setup where we use eight folds for training, one for validation, and one for testing. Each genomic window can serve as an example in a training, validation/tuning, or test set.

#### Model architecture and training

We used the previously optimized DeepSTARR CNN architecture for predicting genome-wide enhancer activity from DNA sequence with minor adaptations^6^. Using the DeepSTARR architecture as a starting point, we performed hyperparameter grid-search to yield best performance on the DNA accessibility validation set of fold01 across the different tissues. The final CNN uses one-hot encoded 1,001 bp long DNA sequence (A = [1,0,0,0], C = [0,1,0,0], G = [0,0,1,0], T = [0,0,0,1]) to predict DNA accessibility signals. The CNN contains four 1D convolutional layers (filters = 256,120,60,60; size = 7,3,3,3; padding = same), each followed by batch normalization, a ReLU non-linearity, and max-pooling (size = 3). After the convolutional layers there are two fully connected layers, with 64 and 256 neurons, respectively, followed by batch normalization, a ReLU non-linearity, and dropout where the fraction is 0.4. The final layer is mapped to the accessibility signal output. Hyperparameters were manually adjusted to yield best performance on the validation set of one cross-validation fold. The models were implemented and trained in Keras (https://keras.io/) from TensorFlow v.1.14.0 (ref. ^56^) using the Adam optimizer^57^ (learning rate = 0.005), mean squared error as loss function, a batch size of 128, and early stopping with patience of five epochs.

To account for variance between different training runs and improve the accuracy and robustness of the models, we trained three replicate models on each held-out test fold (that is, 30 models for each pseudo bulks tissue). After analysing the variance in predictions, and removing the model runs that did not converge (PCC on the test set ≤ 0.1), we averaged the predictions of the replicate models per test set.

#### Model performance

The performance of each model was evaluated on the held-out test chromosomes of each fold. We used the PCC across all bins for a quantitative genome-wide evaluation.

#### Prediction on full *Drosophila* genome

We extracted 1,001 bp sequences tiled across the *Drosophila* dm6 genome (downloaded from https://hgdownload.soe.ucsc.edu/goldenPath/dm6/bigZips/dm6.fa.gz) with a stride of 20 bp using bedtools makewindows (parameters -w 1001 -s 20’) and bedtools getfasta^58^. For each model, we next predicted the accessibility of each genomic window and averaged these per nucleotide to obtain genome-wide coverage.

#### Nucleotide contributions

We used DeepExplainer (the DeepSHAP implementation of DeepLIFT, see refs. ^22–24^ update from https://github.com/AvantiShri/shap/blob/master/shap/explainers/deep/deep_tf.py) to compute contribution scores for all nucleotides in all sequences with respect to the accessibility predictions. We used 100 dinucleotide-shuffled versions of each input sequence as reference sequences. For each sequence, the obtained hypothetical importance scores were multiplied by the one-hot encoded matrix of the sequences to derive the final nucleotide contribution scores. We used one replicate model for each of the 10 folds of cross-validation and averaged the scores for each sequence in each cell type across all the 10 folds. The nucleotide contribution scores were visualized using the ggseqlogo function from the R package *ggseqlogo* (v.0.124).

#### Motif discovery using TF-Modisco

To find important predictive motifs, we ran TF-Modisco (v.0.5.12.0 (ref. ^21^)) on the nucleotide contribution scores of one model fold for each tissue type separately, using the respective accessible regions. We specified the following parameters: sliding_window_size=15, flank_size=5, max_seqlets_per_metacluster=50000 and TfModiscoSeqletsToPatternsFactory(trim_to_window_size=15, initial_flank_to_add=5, final_min_cluster_size=30). We trimmed the PWM motifs by removing flanking positions with an information content lower than 0.4. The TF-Modisco discovered motifs are detailed in Extended Data Fig. 2, the converted PWM logo and the closest match from the transcription factor motif database available at https://github.com/bernardo-de-almeida/motif-clustering^6^ (similarity assessed using TOMTOM^59^ with the following command: tomtom -dist kullback -motif-pseudo 0.1 -text -min-overlap 1).

#### Transcription factor motif analyses across tissues

For the transcription factors that we could assign to the identified motifs, we retrieved their RNA in situ expression data at *Drosophila* embryogenesis stage 13–16 from the Berkeley *Drosophila* Genome Project (BDGP; https://insitu.fruitfly.org/cgi-bin/ex/insitu.pl) and matched their tissue annotation with the tissues used for the sequence-to-accessibility model (see Supplementary Fig. 1b for summary results across tissues and Supplementary Table 1 for full annotation). In addition, we retrieved the transcription factors expression in matched single-cell RNA-seq clusters from the same publication where we retrieved the single-cell ATAC-seq data^16^. The cluster assignment was done through nonnegative least square matrix factorization (see respective publication for details and data; https://shendure-web.gs.washington.edu/content/members/DEAP_website/public/). Transcription factor expression across tissues is displayed in Supplementary Fig. 1c and Supplementary Table 1.

### Deep learning sequence-to-activity models

#### Data preparation

We retrieved the in vivo enhancer activity data from the CAD4 database (available in supplementary table 13 in ref. ^33^), which also includes all enhancer activity data from the Vienna Tiles library (https://enhancers.starklab.org/). For each of the 5 tissues of interest (CNS, epidermis, gut, muscle, brain-specific), we defined sequences as active if they were active between stages 13 and 16 in any of the related tissue annotation terms. CNS: ventral nerve cord, neuroblast of ventral nerve cord primordium, embryonic brain, embryonic central brain, embryonic central brain glial cell, embryonic central brain neuron; epidermis: embryonic dorsal epidermis, embryonic ventral epidermis, embryonic head epidermis, lateral head epidermis, embryonic lateral epidermis, embryonic ventral trunk epidermis, ventral head epidermis, dorsal head epidermis, embryonic epidermis; gut: embryonic hindgut, embryonic midgut chamber, hindgut, embryonic/larval midgut, foregut, midgut interstitial cell; muscle: embryonic/larval somatic muscle, somatic muscle, embryonic somatic muscle, visceral muscle, embryonic/larval visceral muscle, circular visceral muscle fibre, longitudinal visceral muscle fibre, oesophageal visceral muscle, embryonic/larval muscle system, muscle system, dorsal pharyngeal muscle; brain-specific: embryonic brain, embryonic central brain, embryonic central brain glial cell, embryonic central brain neuron AND inactive in the VNC: ventral nerve cord, neuroblast of ventral nerve cord primordium. All the remaining sequences were considered inactive for the respective tissues. For data augmentation, we tiled every sequence in 1,001 bp windows and added also the reverse complement of each original sequence, with the same output, ending up with 176,424 examples (352,848 post-augmentation). Separately for each tissue, we further filtered for active sequences that overlap (minimum overlap of 151 bp) accessibility peaks of the respective tissue to obtain a cleaner positive set. For negative fragments, we selected only at most five different sequences to keep reasonable class imbalances.

#### Cross-validation scheme

We used the same cross-validation folds for training, validation and testing from the accessibility models. Hence, for each fold, the test sets are completely held-out across both stages of training.

#### Model architecture and training

The architecture and weights learned in the first model of the respective tissue were used to initialize this second CNN model to classify DNA sequences based on their activity in vivo, an approach known as transfer learning. For muscle we initialized the model with the visceral muscle accessibility model because it led to a slightly higher performance than initializing with the somatic muscle model (AUPRC of 0.14 vs. 0.12, respectively). We kept all layers trainable and changed the last layer to a sigmoid activation. The models were trained using the Adam optimizer^57^ (with smaller learning rate = 0.0001), binary cross-entropy as loss function, a batch size of 128, and early stopping with patience of twenty epochs.

To account for variance between different training runs and improve the accuracy and robustness of the models, we trained three replicate models on each held-out test fold (that is, 30 models for each of the five tissues, total of 150 models). After analysing the variance in predictions, and removing the model runs that did not converge (area under the curve ≤ 0.7), we averaged the predictions of the replicate models per test set.

#### Model performance

We assessed the model performance of the models of each tissue only on the original, non-augmented Vienna Tiles data, to have a more unbiased set of active and inactive sequences. To have a confident set of positive sequences, we considered as active sequences only the accessibility peaks of the respective tissue that fall (minimum overlap of 201 bp) within tiles active in the respective tissue. As negative sequences we considered both the accessibility peaks that fall (minimum overlap of 201 bp) within tiles inactive in the respective tissue, as well as all other sequences in inactive tiles. We computed the predictions for each sequence using the respective cross-validation set where the sequence is held-out for testing. Using this set of active and inactive tiles per tissue, model performance was accessed using the AUPRC, accuracy, F1-scores (all calculated using confusionMatrix from R package caret v.6.0-90 (ref. ^60^)), and by estimating the positive predictive value (percentage of validated active sequences among all positive predictions) at different prediction thresholds.

We also evaluated the sequence-to-activity models for known tissue-specific enhancers in marker gene loci of each tissue (enhancers in our database present in ±50kb from the transcription start site): *elav* (CNS), *grh* (epidermis), *GATAe* (gut), *Mef2* (muscle) and *tll* (brain) (Extended Data Fig. 6). There were no enhancers in epidermis *wg* locus, so we replaced it by the epidermis marker gene *grh*.

#### Comparison with different model initializations

For each of the five tissues, we compared the performance of the fine-tuned models with transfer learning with (1) models pre-trained on DNA accessibility of a different tissue (salivary gland, since it has very different profiles when compared with the five tissues of interest; see Extended Data Fig. 1a) and (2) models directly trained on the in vivo enhancer activity data starting from random initialization (no fine-tuning). Model architecture, training and cross-validation schemes, as well as performance evaluation were identical to the ones described above for the main model.

#### Nucleotide contributions

Same as described for the accessibility models above.

### Computational design of *Drosophila* enhancers

Three billion random 501 bp DNA sequences were generated in bash with the following code: cat /dev/urandom | tr -dc ‘ACGT’ | fold -w 501 | head -n 3000000000 and flanked left and right with random 250 bp sequences to obtain 1,001 bp long sequences. We predicted these sequences’ activities and accessibilities with one replicate model per tissue (taking less than 10 min for 100,000 sequences per model on a single CPU) until we had ~15,000 sequences predicted to be specifically active and accessible in the five target tissues (CNS, epidermis, gut, muscle, brain). From the top 3,000 candidates, we randomly sampled 100 and computed the nucleotide contribution scores for visual inspection of motif content and arrangement, alongside the candidates’ prediction scores. We made sure the predicted activity is independent of the ±250 bp flanks by predicting the activity of each of the selected middle 501-bp sequences with 100 different ±251 bp flanks. Based on this combined information, we then manually selected eight candidates per tissue for testing in vivo (Supplementary Table 2). We searched the candidate synthetic enhancers against the *Drosophila* genome (taxid:7227) using Blastn via NIH NCBI Blast https://blast.ncbi.nlm.nih.gov/Blast.cgi with default parameters, except for word size of 7 (smallest and thus most sensitive setting) and expectation value (E value) threshold of 10. Two candidates (active muscle_synth5 and inactive gut_synth9) had matches with E values of 0.032, which corresponds to 22/501 bp shared sequence; no other candidate had matches with E value ≤ 0.1.

#### Distribution of prediction scores in random sequences

We scored 100,000 random 1,001 bp sequences with the sequence-to-activity transfer-learned models as well as activity models directly trained on the in vivo enhancer activity data starting from random initialization. We used the same replicate model of the random sequence selection above for each tissue. We calculated the percentiles of the final 40 synthetic enhancers in the distributions of the two models in each tissue.

#### Final enhancer activity scores of the selected 40 candidates

To obtain the final expected enhancer activities (= final scores) for the selected 40 candidates, we placed the 501 bp sequences of each candidate within the ±250 bp flanks of the actual reporter construct and scored the resultant 1-kb sequences with the transfer learning enhancer-to-activity models of each tissue. We used one replicate model for each of the ten folds of cross-validation and averaged the predictions across folds.

#### Nucleotide contributions

Same as described for the accessibility models above but using the 501 bp synthetic sequences flanked by the actual sequence of the plasmid where they were inserted for testing in vivo.

### Cloning of synthetic *Drosophila* enhancers

The 501-bp synthetic sequences (designed above; Supplementary Table 2) were ordered from Twist Bioscience flanked by 20-bp linkers for Gibson assembly (5′, GAATTGGGAATTCGTTAACA; 3′, TGGTCTAGAGCCCGGGCGAA). Sequences were cloned upstream of a minimal hsp70 promoter driving a *lacZ* reporter gene in an attB-containing plasmid^33^, linearized with BglII using Gibson Assembly. Plasmids were verified by Sanger sequencing. 27 µg per plasmid (45 µl; 600 ng µl^−1^) were sent to BestGene for injection in *Drosophila* embryos (integration site: http://flybase.org/reports/FBst0024482.html) and positive transformants were selected. All constructs were injected into embryos according to standard methods and inserted into the attP landing site line M{3×P3-RFP.attP′}ZH-51C via PhiC31 integrase insertion, yielding integration at chromosomal position 51C1.

Such reporter systems provide an opportunity to measure enhancer activities and the enhancers’ spatio-temporal activity patterns in a constant and controlled environment^4,33,61^. The hsp70 core promoter has been widely used for transgene expression and enhancer testing (for example, ref. ^33^) and functions highly similarly as other developmental promoters (for example, DSCP)^62^. While controlled reporter systems differ from endogenous gene regulation, we previously found that 82% of the enhancer–activity patterns reflect the enhancers’ endogenous activities^4^.

### Embryo fixation for imaging

Embryos of the respective genotypes were washed off collection plates into a collection bottle with a mesh at the bottom using paintbrushes and water. Afterwards, the embryos were dechorionated for 2 min in 50% bleach. Following dechorionation, embryos were washed extensively with water and were collected eventually on the mesh of the collection bottle with 1x PBT (PBS, 0.1% Triton X-100). After drying the embryos on the mesh on a piece of tissue paper they were transferred into a 1.5-ml reaction tube with 1 volume fixation solution (4% (v/v) formaldehyde in PBS) and 1 volume heptane. Embryos were fixed for 20 min on a horizontal shaker at 500 rpm. To devitellinize the embryos the fixation solution was aspirated and 1 volume methanol was added to the tube, followed by extensive shaking. The heptane phase and excess methanol were removed, leaving the devitellinized embryos at the bottom of the tube. Embryos were washed three times with methanol and stored in methanol or ethanol at −20 °C.

### FISH in *Drosophila* embryos

Whole-mount *Drosophila* RNA in situ hybridization experiments were carried out as described previously^63^. Digoxigenin-labelled RNA anti-sense probes for *elav*, *wg*, *GATAe*, *mef2* as well as *tll* were prepared from corresponding EST clones from the DGRC collections (*Drosophila* Genomics Resource Center (NIH Grant 2P40OD010949)) using the DIG labelling mix (Roche, 11175033910) and T3, T7 or SP6 RNA polymerase (Roche) according to the manufacturer’s instructions. Fluorescein-labelled RNA anti-sense probe for *lacZ* was prepared from a PCR fragment that has been amplified from a pGEMT easy plasmid containing the *lacZ* gene using the Fluorescein labelling mix (Roche, 11685619910) and T7 RNA polymerase (Roche) according to the manufacturer’s instructions. mRNA expression was visualized from these probes using anti-Digoxigenin-Peroxidase (Roche 11633716001) and anti-Fluorescein-Peroxidase (Roche 11426346910) (all antibodies diluted 1:2,000) coupled with the TSA Plus Cyanine 3 (Akoya Biosciences, NEL744001KT) and TSA Plus Fluorescein (Akoya Biosciences, NEL741001KT) kits.

### Qualitative visual pattern assessment and imaging of representative FISH-stained embryos

Two-hundred to three-hundred double FISH-stained embryos with the respective genetic background were mounted in ProLong Gold mounting medium with DAPI (ThermoFisher Scientific P36931) and scored individually for *lacZ* reporter expression in embryonic stage 13-14. If a synthetic enhancer-driven *lacZ* expression pattern was observed in all homozygous embryos in a reproducible manner, the enhancer was scored as active. For these, one representative homozygous embryo was selected and a *z* stack (1 µm step size, between 7–12 slices per embryo) was imaged on a Zeiss LSM 880 Airyscan Fast confocal microscope using a Plan Apochromat 20×/0.8 objective. For visualization of the enhancer-driven reporter expression in relation to the tissue-specific marker gene expression, a maximum projection of the *z* stack was performed in Fiji^64^.

### Quantification of tissue-specific enhancer activity in FISH-stained embryos

For the quantification of enhancer activity in the predicted tissue we analysed its reporter expression pattern in spatial relation to the respective tissue-specific marker expression and calculated a PCC. For this purpose, we imaged *z*-stacks (1 µm step size, between 7–12 slices per embryo) of 4 double FISH-stained embryos of the respective genotype with low-resolution (256 × 256 Pixel) on a Zeiss LSM 880 Airyscan Fast confocal microscope using a Plan Apochromat 20×/0.8 objective. Subsequently, we calculated the PCC between the two channels with Fiji^64^ utilizing the JACoP plugin^65^ with standard parameters. As controls we used either double FISH-stained embryos that showed no reporter expression or embryos double FISH-stained for the unrelated *Myosin heavy chain* (*MHC*, muscle) and *cacophony* (*cac*, CNS) genes.

### Statistics and data visualization

All statistical calculations and graphical displays have been performed in R statistical computing environment (v.3.5.1 (ref. ^66^)) and using the R package ggplot2 (v.3.2.1 (ref. ^67^)). Coverage data tracks have been visualized in the UCSC Genome Browser^68^ and used to create displays of representative genomic loci. In all boxplots, the central line denotes the median, the box encompasses 25th to 75th percentile (interquartile range) and the whiskers extend to 1.5× interquartile range.

### Reporting summary

Further information on research design is available in the Nature Portfolio Reporting Summary linked to this article.

## Online content

Any methods, additional references, Nature Portfolio reporting summaries, source data, extended data, supplementary information, acknowledgements, peer review information; details of author contributions and competing interests; and statements of data and code availability are available at 10.1038/s41586-023-06905-9.

### Supplementary information

Supplementary Information This file contains Supplementary Figs. 1 and 2. Supplementary Fig. 1: Transcription factor motifs predictive of DNA accessibility. Supplementary Fig. 2: Comparison between transfer learning and random initialization enhancer activity models.

Reporting Summary

Supplementary Table 1 Transcription factor motifs predictive of DNA accessibility. Table with motifs discovered by TF-Modisco across the different tissues, including the predicted transcription factor, and the tissues where the motif was discovered by TF-Modisco (including the motif ID). For the transcription factors that we could assign to the identified motifs, expression values across matched single-cell RNA-seq clusters of the respective tissues are shown. Final column contains the expression annotation of the transcription factor at stage 13–16 from RNA in situ experiments from the BDGP (https://insitu.fruitfly.org/cgi-bin/ex/insitu.pl).

Supplementary Table 2 Results of in vivo validation of candidate sequences in the *Drosophila* embryo. Detailed information about each candidate sequence, including the respective DNA sequence, the results of in vivo validation and detailed annotation of expression results, predicted scores with the enhancer activity models from the respective tissue, and their percentiles among other 100,000 randomly generated sequences.

Supplementary Table 3 Sequence splits used for tenfold cross-validation analysis.

## Data Availability

The transcription factor motif database is available at https://github.com/bernardo-de-almeida/motif-clustering. The final pre-trained accessibility and enhancer activity models, as well as the data used to train and evaluate the models, are available at 10.5281/zenodo.8011697. All reporter DNA constructs and transgenic flies for active synthetic enhancers are available from the Vienna *Drosophila* Resource Center (VDRC) at https://shop.vbc.ac.at/vdrc_store/vdrc-fly-stocks/other-resources/a-stark-stocks-as-stock.html.

## Code availability

Code used to train the models and to make predictions on new sequences is available on GitHub (https://github.com/bernardo-de-almeida/DeepSTARR_embryo).
