# Supplementary material for: Targeted design of synthetic enhancers for selected tissues in the Drosophila embryo
Source: Nature. 2023 Dec 12;626(7997):207–11. doi: 10.1038/s41586-023-06905-9 (PMC10830412; doi:10.1038/s41586-023-06905-9)
Supplement: Supplementary file 1 — This file contains Supplementary Figs. 1 and 2. Supplementary Fig. 1: Transcription factor motifs predictive of DNA accessibility. Supplementary Fig. 2: Comparison between transfer learning and random initialization enhancer activity models. [file 41586_2023_6905_MOESM1_ESM.pdf]

nature portfolio

https://doi.org/10.1038/s41586-023-06905-9

Supplementary information

Targeted design of synthetic enhancers for selected tissues in the *Drosophila* embryo

In the format provided by the authors and unedited

Supplementary Information Guide

Targeted design of synthetic enhancers for selected tissues in the *Drosophila* embryo

Bernardo P. de Almeida[1,2,†], Christoph Schaub[3], Michaela Pagani[1], Stefano Secchia[3], Eileen E. M. Furlong[3], Alexander Stark[1,4*]

[1] Research Institute of Molecular Pathology (IMP), Vienna BioCenter (VBC), Vienna, Austria
[2] Vienna BioCenter PhD Program, Doctoral School of the University of Vienna and Medical University of Vienna, Vienna, Austria
[3] European Molecular Biology Laboratory (EMBL), Genome Biology Unit, Heidelberg, Germany
[4] Medical University of Vienna, Vienna BioCenter (VBC), Vienna, Austria
[†] Current affiliation: InstaDeep, Paris, France

*Corresponding author. Email: stark@starklab.org

Table of Contents

Supplementary Figures 3

Supplementary Fig. 1. TF motifs predictive of DNA accessibility. 3

Supplementary Fig. 2 Comparison between transfer-learning and random-initialization enhancer activity models. 5

Supplementary Tables 7

Table S1. TF motifs predictive of DNA accessibility. 7

Table S2. Results of *in vivo* validation of candidate sequences in the *Drosophila* embryo. 7

Table S3. Sequence splits used for 10-fold cross-validation analysis. 7

Supplementary Figures

Supplementary Fig. 1. TF motifs predictive of DNA accessibility.

A TF motifs discovered by TF-Modisco

TF motif ID	*Drosophila* TF	Discovered in CNS	Discovered in brain	Discovered in epidermis	Discovered in midgut	Discovered in somatic muscle	Discovered in visceral muscle
Trl/1, Trl/2	Trl	yes (motif 2)	yes (motif 1)	yes (motif 2 & 4)	yes (motif 2)	yes (motif 1)	yes (motif 2)
DRE/1, DRE/2	Dref, BEAF-32	yes (motif 3)	yes (motif 2)	yes (motif 5)	yes (motif 3)	yes (motif 2)	yes (motif 1)
Ohler1	?	yes (motif 5)	yes (motif 3)	yes (motif 6)	yes (motif 7 & 19)	yes (motif 6)	yes (motif 3)
CA-repeat/1, CA-repeat/2	?	yes (motif 6)	yes (motif 6)	yes (motif 8)	yes (motif 6)	yes (motif 3)	yes (motif 5)
Ebox/CAGCTG/3 (Ohler5)	?	yes (motif 10)	yes (motif 9)	yes (motif 9)	yes (motif 8)	yes (motif 8)	yes (motif 9)
HSF/6	Hsf	yes (motif 13)	yes (motif 17)	yes (motif 17)	yes (motif 16)	yes (motif 17)	yes (motif 16)
CTCF	CTCF	yes (motif 12)	yes (motif 14)	yes (motif 14 & 16)	no	yes (motif 14)	no
grh/3	grh	yes (motif 14)	yes (motif 13)	yes (motif 1)	yes (motif 17)	no	no
Mef2/1, Mef2/2	Mef2	no	no	yes (motif 11)	yes (motif 9)	yes (motif 4)	yes (motif 10 & 14)
Unknown6	?	yes (motif 11)	no	no	no	yes (motif 12)	yes (motif 7)
kni/1	kni	no	yes (motif 8)	yes (motif 7)	yes (motif 5)	no	no
Ebox/CATGTG/twi, Ebox/CATATG/CATCTG	twi	no	yes (motif 18)	no	no	yes (motif 10)	yes (motif 13)
NHP6A/IRX	?	no	no	yes (motif 20)	no	yes (motif 16)	yes (motif 24)
su(Hw)/2	su(Hw)	no	no	no	yes (motif 15)	yes (motif 18)	yes (motif 17)
FOX/CG11152	fd102C	no	yes (motif 7)	no	no	yes (motif 15)	no
AGGGG	ttk, Kr	yes (motif 1)	yes (motif 4)	no	no	no	no
Dip3/PAX4	?	yes (motif 9)	no	no	no	yes (motif 19)	no
vfl (zld)	zld	no	yes (motif 11)	yes (motif 10)	no	no	no
Ohler6	?	no	yes (motif 15)	no	no	yes (motif 7)	no
Unknown4	?	no	no	yes (motif 18)	no	no	yes (motif 22)
Aef1/5	Aef1, CG4360	no	no	yes (motif 19)	no	no	yes (motif 15)
NRF1	?	yes (motif 7)	no	no	no	no	no
GCCG	?	no	yes (motif 10)	no	no	no	no
CG31782	CG31782	no	yes (motif 16)	no	no	no	no
ACA/1	?	no	yes (motif 20)	no	no	no	no
ZIC5	?	no	no	yes (motif 3)	no	no	no
GATA/2	GATAd, GATAe	no	no	no	yes (motif 1 & 10 & 20)	no	no
GATA/MECOM/2	GATAd, GATAe	no	no	no	yes (motif 11)	no	no
SPT3/4	?	no	no	no	yes (motif 4)	no	no
Ohler7	?	no	no	no	yes (motif 12)	no	no
DMRT2	?	no	no	no	yes (motif 13)	no	no
ZNF263/2	?	no	no	no	yes (motif 14)	no	no
SFL1	?	no	no	no	yes (motif 18)	no	no
ci	ci	no	no	no	no	yes (motif 9)	no
E2F/brk	brk	no	no	no	no	yes (motif 11)	no
EGR/2	?	no	no	no	no	yes (motif 20)	no
AT−tract/4	?	no	no	no	no	yes (motif 21)	no
Zscan4	?	no	no	no	no	yes (motif 22)	no
FOX/2	bin, fd102C	no	no	no	no	no	yes (motif 6)
CG−tract/4	?	no	no	no	no	no	yes (motif 11)
ETF/1	?	no	no	no	no	no	yes (motif 12)
YY1/1	?	no	no	no	no	no	yes (motif 18)
Mad/2	Mad	no	no	no	no	no	yes (motif 19)
CG32830/br/vvl	ab, br, vvl	no	no	no	no	no	yes (motif 20)
TATA/NHP6	?	no	no	no	no	no	yes (motif 21)

B TF expression by *in-situ* (BDGP database)

TF motif ID	*Drosophila* TF	Expressed in CNS	Expressed in brain	Expressed in epidermis	Expressed in midgut	Expressed in somatic muscle	Expressed in visceral muscle
Trl/1, Trl/2	Trl	yes	yes	yes	yes	yes	yes
DRE/1, DRE/2	Dref, BEAF-32	yes	yes	yes	yes	yes	yes
HSF/6	Hsf	yes	yes	yes	yes	yes	yes
CTCF	CTCF	yes	yes	no	no	no	no
grh/3	grh	no	no	yes	no	no	no
Mef2/1, Mef2/2	Mef2	no	no	no	no	yes	yes
kni/1	kni	no	no	no	no	no	no
Ebox/CATGTG/twi, Ebox/CATATG/CATCTG	twi	no	no	no	no	yes	yes
	su(Hw)	yes	yes	yes	yes	yes	yes
	fd102C	no	yes	no	no	no	no
AGGGG	ttk, Kr	no, yes	no, yes	yes, yes	yes, yes	no	no
vfl (zld)	zld	yes	yes	yes	no	yes	no
Aef1/5	Aef1, CG4360	yes	yes	yes	yes	yes	yes
CG31782	CG31782	no	no	no	no	no	no
GATA/2	GATAd, GATAe	yes, no	yes, no	yes, no	yes, yes	yes, no	yes, no
GATA/MECOM/2	GATAd, GATAe	yes, no	yes, no	yes, no	yes, yes	yes, no	yes, no
ci	ci	no	no	yes	no	no	no
E2F/brk	brk	no	no	yes	yes	yes	yes
FOX/2	bin, fd102C	no, no	no, yes	no, no	no, no	no, no	yes, no
Mad/2	Mad	no	no	no	yes	no	no
CG32830/br/vvl	ab, br, vvl	no, yes, no	no, yes, no	yes, no, yes	no, yes, no	yes, no, no	yes, no, no

C TF expression in matched single-cell RNA-seq clusters

TF motif ID	*Drosophila* TF	CNS	brain	epidermis	midgut	somatic muscle	visceral muscle
Trl/1, Trl/2	Trl	0,46	0,73	0,5	**0,86**	0,73	0,76
DRE/1, DRE/2	Dref, BEAF-32	0,03, 0,04	0,04, **0,06**	0,02, 0,02	0,03, 0,06	**0,07**, 0,02	0,04, 0,03
HSF/6	Hsf	**0,16**	0,13	0,11	0,14	0,13	**0,16**
CTCF	CTCF	0,01	0,02	0,01	**0,03**	0,02	0,02
grh/3	grh	0,57	0,59	**1,65**	0,43	0,62	0,71
Mef2/1, Mef2/2	Mef2	0,39	0,44	0,36	0,41	**1,34**	0,75
kni/1	kni	0,05	0,07	**0,15**	0,04	0,08	0,05
Ebox/CATGTG/twi, Ebox/CATATG/CATCTG	twi	0,02	0,01	0,02	0,01	**0,03**	**0,03**
AGGGG	ttk, Kr	1,9, 0,08	1,04, **0,21**	**2,03**, 0,06	1,37, 0,06	1,54, 0,04	1,4, 0,1
vfl (zld)	zld	**0,56**	0,49	0,43	0,16	0,4	0,42
Aef1/5	Aef1, CG4360	0, 0,12	0,05, 0,09	**0,08, 0,14**	**0,08, 0,14**	0,05, 0,09	0,05, 0,12
CG31782	CG31782	NA	NA	NA	NA	NA	NA
GATA/2	GATAd, GATAe	**0,1**, 0,07	0,09, 0,08	0,08, 0,07	0,09, **0,47**	0,09, 0,1	0,07, 0,07
GATA/MECOM/2	GATAd, GATAe	**0,1**, 0,07	0,09, 0,08	0,08, 0,07	0,09, **0,47**	0,09, 0,1	0,07, 0,07
ci	ci	0,3	0,26	**0,46**	0,26	0,39	0,44
E2F/brk	brk	**0,17**	0,04	0,09	0,07	0,06	0,06
FOX/2	bin, fd102C	0, NA	0,02, NA	0,01, NA	0, NA	0.04, NA	**0.05, NA**
Mad/2	Mad	0,17	0,21	0,2	0,2	**0,22**	0,19
CG32830/br/vvl	ab, br, vvl	0,8, 0,06, **0,97**	1,07, **0,64**, 0,5	**2,38**, 0,1, 0,57	0,85, 0,13, 0,31	1,78, 0,15, 0,31	1,35, 0,3, 0,39

A) Table with motifs discovered by TF-Modisco across the different tissues. Motifs from Extended Data Fig. 2. Cells with "yes" are highlighted in color and have the respective motif ID from TF-Modisco. **B)** RNA *in situ* expression of TFs that we could assign to the identified motifs in (A) across tissues. Data from the Berkeley Drosophila Genome Project (BDGP; https://insitu.fruitfly.org/cgi-bin/ex/insitu.pl; see Table S1 for full annotation). Cells with "yes" are highlighted in color. **C)** TF expression values across matched single-cell RNA-seq clusters of the respective tissues. For each TF, the tissue with the highest expression is highlighted in color and bold.

Supplementary Fig. 2 Comparison between transfer-learning and random-initialization enhancer activity models.

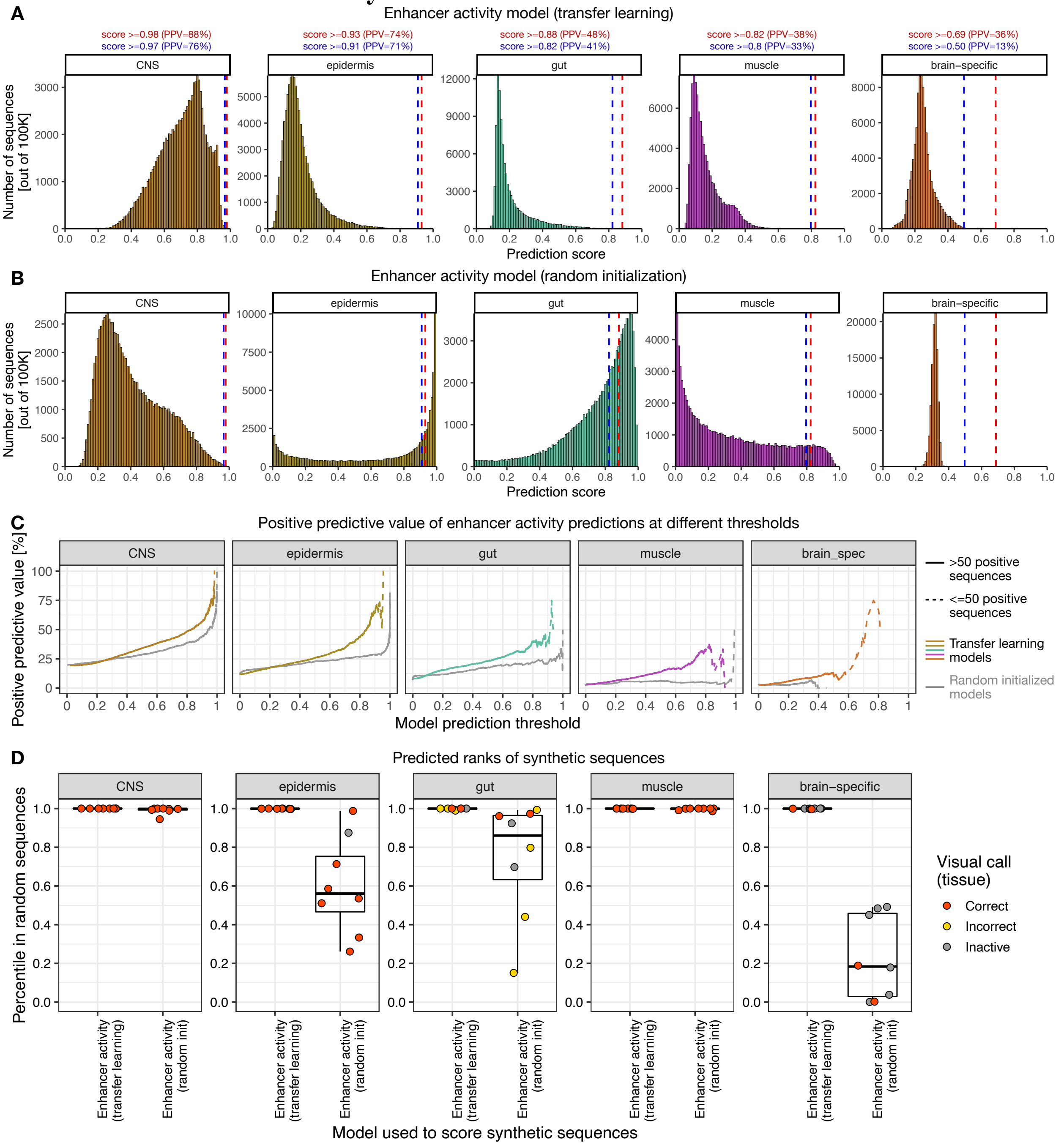

A-B) Histograms of prediction scores for 100,000 (100K) random sequences by the enhancer activity models with transfer learning (A) or the random initialized models (B) of each tissue. For each tissue, the Positive Predictive Value (PPV) and respective predictive score thresholds (vertical dashed lines) from Fig. 1A are shown. **C)** Positive predictive value of enhancer activity predictions at different thresholds for the models with transfer learning (colored) or models based on random initialization (grey) for each tissue. For each threshold [X-axis, 0-1], the percentage of active sequences among all positive predictions is shown [Y-axis]. Solid lines indicate percentages calculated based on more than 50 positive sequences, while dashed lines represent less confident

estimates based on smaller numbers. **D)** Percentile of the 40 synthetic candidate sequences among other 100,000 generated random sequences as scored by the tissue-specific enhancer activity models using transfer learning or using random initialization. Individual candidate sequences (dots) are colored based on their validated *in vivo* activity: correct tissue expression, incorrect tissue expression and inactive. Boxplots using the values from the 8 candidates per tissue are also shown. The boxplots mark the median, upper and lower quartiles and 1.5× interquartile range (whiskers).

Supplementary Tables

Table S1. TF motifs predictive of DNA accessibility.
Table with motifs discovered by TF-Modisco across the different tissues, including the predicted TF, and the tissues where the motif was discovered by TF-Modisco (including the motif ID). For the TFs that we could assign to the identified motifs, expression values across matched single-cell RNA-seq clusters of the respective tissues are shown. Final column contains the expression annotation of the TF at stage 13-16 from RNA *in situ* experiments from the Berkeley Drosophila Genome Project (BDGP; https://insitu.fruitfly.org/cgi-bin/ex/insitu.pl).

Table S2. Results of *in vivo* validation of candidate sequences in the *Drosophila* embryo.
Detailed information about each candidate sequence, including the respective DNA sequence, the results of *in vivo* validation and detailed annotation of expression results, predicted scores with the enhancer activity models from the respective tissue, and their percentiles among other 100,000 randomly generated sequences.

Table S3. Sequence splits used for 10-fold cross-validation analysis.
