# Supplementary material for: Targeted design of synthetic enhancers for selected tissues in the Drosophila embryo
Source: Nature. 2023 Dec 12;626(7997):207–11. doi: 10.1038/s41586-023-06905-9 (PMC10830412; doi:10.1038/s41586-023-06905-9)
Supplement: Supplementary file 2 — Reporting Summary [file 41586_2023_6905_MOESM2_ESM.pdf]

nature portfolio

Corresponding author(s): Alexander Stark

Last updated by author(s): 05/11/2023

Reporting Summary

Nature Portfolio wishes to improve the reproducibility of the work that we publish. This form provides structure for consistency and transparency in reporting. For further information on Nature Portfolio policies, see our Editorial Policies and the Editorial Policy Checklist.

Statistics

For all statistical analyses, confirm that the following items are present in the figure legend, table legend, main text, or Methods section.

n/a	Confirmed	
☐	☒	The exact sample size (*n*) for each experimental group/condition, given as a discrete number and unit of measurement
☐	☒	A statement on whether measurements were taken from distinct samples or whether the same sample was measured repeatedly
☐	☒	The statistical test(s) used AND whether they are one- or two-sided<br>*Only common tests should be described solely by name; describe more complex techniques in the Methods section.*
☒	☐	A description of all covariates tested
☐	☒	A description of any assumptions or corrections, such as tests of normality and adjustment for multiple comparisons
☐	☒	A full description of the statistical parameters including central tendency (e.g. means) or other basic estimates (e.g. regression coefficient) AND variation (e.g. standard deviation) or associated estimates of uncertainty (e.g. confidence intervals)
☐	☒	For null hypothesis testing, the test statistic (e.g. *F*, *t*, *r*) with confidence intervals, effect sizes, degrees of freedom and *P* value noted<br>*Give P values as exact values whenever suitable.*
☒	☐	For Bayesian analysis, information on the choice of priors and Markov chain Monte Carlo settings
☒	☐	For hierarchical and complex designs, identification of the appropriate level for tests and full reporting of outcomes
☐	☒	Estimates of effect sizes (e.g. Cohen's *d*, Pearson's *r*), indicating how they were calculated

Our web collection on statistics for biologists contains articles on many of the points above.

Software and code

Policy information about availability of computer code

Data collection	NA
Data analysis	Code used to train the models as well as to make predictions on new sequences will is available on GitHub (https://github.com/bernardo-de-almeida/DeepSTARR_embryo).<br><br>Peak calling: MASC2<br>Extracting DNA sequences of genomic windows across the genome: bedtools version 2.27.1.<br><br>Deep learning models were implemented and trained in Keras (v.2.2.4) (with TensorFlow v.1.14.0) using the Adam optimizer. DeepExplainer (the DeepSHAP implementation of DeepLIFT; update from version in https://github.com/AvantiShri/shap/blob/master/shap/explainers/deep/deep_tf.py) was used to compute contribution scores. TF–Modisco (v.0.5.12.0) used the contribution scores to derive TF motifs.<br><br>Sequence alignment was done using Blastn via NIH NCBI Blast https://blast.ncbi.nlm.nih.gov/Blast.cgi.<br><br>Coverage data tracks have been visualized in the UCSC Genome Browser https://genome.ucsc.edu/.<br><br>All statistical calculations and graphical displays have been performed in R statistical computing environment (v.3.5.1) and using the R package ggplot2 (v.3.2.1).

For manuscripts utilizing custom algorithms or software that are central to the research but not yet described in published literature, software must be made available to editors and reviewers. We strongly encourage code deposition in a community repository (e.g. GitHub). See the Nature Portfolio guidelines for submitting code & software for further information.

Data

Policy information about availability of data

All manuscripts must include a data availability statement. This statement should provide the following information, where applicable:

- Accession codes, unique identifiers, or web links for publicly available datasets
- A description of any restrictions on data availability
- For clinical datasets or third party data, please ensure that the statement adheres to our policy

The Drosophila embryo scATAC-seq data used in this study is publicly available at https://shendure-web.gs.washington.edu/content/members/DEAP_website/public/. We retrieved sci-ATAC-seq3 mapped reads (dm6) from each of the 18 tissue pseudo-bulk (i.e. mapped reads from all cells combined) at the 10-12 hours timepoint from ref. (12) (downloaded from https://shendure-web.gs.washington.edu/content/members/DEAP_website/public/ on Feb. 1st 2022, BAM files available upon request). The Drosophila dm6 genomic sequence was downloaded as a fasta file from https://hgdownload.soe.ucsc.edu/goldenPath/dm6/bigZips/dm6.fa.gz. The TF motif database is available at https://github.com/bernardo-de-almeida/motif-clustering. TF expression data was retrieved from BDGP (RNA in situ; https://insitu.fruitfly.org/cgi-bin/ex/insitu.pl) and https://shendure-web.gs.washington.edu/content/members/DEAP_website/public/ (single-cell RNA-seq). The enhancer activity data from the Vienna Tiles library is available at https://enhancers.starklab.org/, and the enhancer activity database CAD4 is available from REF PMID: 29539636 (Table S13). The final pre-trained accessibility and enhancer activity models, as well as the data used to train and evaluate the models, can be found on zenodo at https://doi.org/10.5281/zenodo.8011697.

Research involving human participants, their data, or biological material

Policy information about studies with human participants or human data. See also policy information about sex, gender (identity/presentation), and sexual orientation and race, ethnicity and racism.

Reporting on sex and gender	This study did not involve human participants.
Reporting on race, ethnicity, or other socially relevant groupings	NA
Population characteristics	NA
Recruitment	NA
Ethics oversight	NA

Note that full information on the approval of the study protocol must also be provided in the manuscript.

Field-specific reporting

Please select the one below that is the best fit for your research. If you are not sure, read the appropriate sections before making your selection.

☒ Life sciences ☐ Behavioural & social sciences ☐ Ecological, evolutionary & environmental sciences

For a reference copy of the document with all sections, see nature.com/documents/nr-reporting-summary-flat.pdf

Life sciences study design

All studies must disclose on these points even when the disclosure is negative.

Sample size	Sample size is not relevant for the machine learning models presented in this work. For imaging of FISH-stained embryos, 200-300 of FISH stained embryos with the respective genetic background were used, of which representative images were chosen. This number of embryos is sufficient to analyze the consistency of the enhancer activity pattern.
Data exclusions	No data was excluded.
Replication	The deep learning models were replicated through 10-fold cross-validation to asses their performance, with similar results. For imaging of the activity of synthetic enhancers, 200-300 of FISH-stained embryos with the respective genetic background were used and the activity pattern was consistent across them.
Randomization	Not relevant because the samples were not grouped.
Blinding	Researchers were not blind to the identity of the genetic backgrounds of the embryos. For the remaining analyses, it is not relevant because the samples were not grouped.

Reporting for specific materials, systems and methods

We require information from authors about some types of materials, experimental systems and methods used in many studies. Here, indicate whether each material, system or method listed is relevant to your study. If you are not sure if a list item applies to your research, read the appropriate section before selecting a response.

Materials & experimental systems

n/a	Involved in the study
☐	☒ Antibodies
☒	☐ Eukaryotic cell lines
☒	☐ Palaeontology and archaeology
☐	☒ Animals and other organisms
☒	☐ Clinical data
☒	☐ Dual use research of concern
☒	☐ Plants

Methods

n/a	Involved in the study
☒	☐ ChIP-seq
☒	☐ Flow cytometry
☒	☐ MRI-based neuroimaging

Antibodies

Antibodies used	Anti-Digoxigenin-Peroxidase, Roche #11633716001<br>Anti-Fluorescein-Peroxidase, Roche #11426346910
Validation	Commercial antibodies validated in a previous paper (Schor et al, Current Biology 2018).

Animals and other research organisms

Policy information about studies involving animals; ARRIVE guidelines recommended for reporting animal research, and Sex and Gender in Research

Laboratory animals	Drosophila embryos from FlyC31 strains (genotype M{3xP3-RFP.attP'}ZH-51C) were collected at BestGene Inc. and imaged at in embryonic stage 13-14.
Wild animals	No wild animals were used
Reporting on sex	Mixed male and female
Field-collected samples	No filed-collected samples were used
Ethics oversight	No approval required

Note that full information on the approval of the study protocol must also be provided in the manuscript.
